# Video‐Sound Recording Devices Reveal Multiple Drivers of Nocturnal Vocalizations in Tibetan Macaques

**DOI:** 10.1002/ece3.72587

**Published:** 2025-12-12

**Authors:** Xin Gao, Tong Zhang, Jingjing Wang, Dongxin Yang, Xue Chen, Peipei Yang, Wenbo Li, Binghua Sun, Dongpo Xia, Jinhua Li, Xi Wang

**Affiliations:** ^1^ School of Resources and Environmental Engineering Anhui University Hefei China; ^2^ International Collaborative Research Center for Huangshan Biodiversity and Tibetan Macaque Behavioral Ecology Hefei China; ^3^ School of Life Sciences Anhui University Hefei China; ^4^ School of Life Sciences Hefei Normal University Hefei China; ^5^ Behavioral Ecology & Sociobiology Unit German Primate Center Göttingen Germany

**Keywords:** diurnal primate, ecological factors, nocturnal vocalization, social behavior, Tibetan macaque

## Abstract

Nocturnal vocalizations not only reflect communications at night, but also reveal adaptive strategies of animals. Previous research focused on the vocalizations of nocturnal species, with little attention to nocturnal vocalizations in diurnal animals. Furthermore, sound studies traditionally employ an active acoustic monitoring approach which is limited in terms of the range of sound collection and is not combined with video data. To fill in these gaps, we studied the nocturnal vocalization of diurnal primates, Tibetan macaques ( *Macaca thibetana* ) in Huangshan, China by 4G infrared cameras and passive acoustic monitoring devices. Results showed that Tibetan macaques had a possible bimodal distribution of nocturnal vocalization count, with peaks occurring at 18:00–19:00 and 21:00–23:00. In terms of socio‐biological factors, males or individuals with lower social centrality exhibited more vocalization count, and kinship also promoted the emission of vocalization, whereas vocalization was negatively correlated with age. As for ecological factors, wind direction significantly affected the vocal activity level. Vocal activity level was correlated with environmental variables, showing lower values under high wind speed, and higher values under low temperature and high humidity, with additional interactions among climatic factors such as temperature and precipitation. An analysis of the behavioral context within 10 s before and after vocalizations found that, diverse social behaviors were frequent around 18:00, but affiliative behaviors were most frequent between 21:00 and 22:00. Based on video‐sound recording devices, we first revealed the pattern of nocturnal vocalization in *Macaca thibetana* and its factors. Nocturnal vocalizations of diurnal primates likely serve to maintain group cohesion, manage conflicts, adapt to environmental pressures, and facilitate group integration, reflecting the multifaceted strategies by primates for survival and social organization.

## Graphical abstract

This study investigates the nocturnal vocal behavior of Tibetan macaques in Huangshan, China, using 4G infrared cameras and passive acoustic monitoring (PAM). Findings reveal a bimodal vocalization pattern (18:00–19:00 and 21:00–23:00), influenced by sex, age, social centrality, kinship, and ecological factors such as wind direction, temperature, and humidity. Nocturnal vocalizations play a multifunctional role in maintaining group cohesion, managing social conflicts, adapting to environmental challenges, and integrating peripheral individuals.

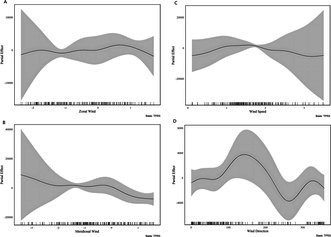

## 1 Introduction

Daily and nocturnal activity patterns have gradually become a focus in animal behavior and ecology, as mammals display diverse activity rhythms including diurnal, nocturnal, crepuscular, and cathemeral patterns (Refinetti 2008). These variations in activity patterns illustrate not only the adaptation of different species to their natural environments but also their physiological needs and social structures. Diurnal animals typically rely on vision for foraging and performing social interactions during daylight, whereas nocturnal species depend more on their auditory and olfactory systems to navigate their environment and avoid predation at night (Refinetti 2008). These differences in sensory reliance are related to habitat characteristics, food availability, and predation pressure, which in turn influence resource competition, dominance style, and the social structure of a species. However, growing evidence suggests that traditionally diurnal species also exhibit nocturnal activity under specific conditions. For instance, mammals, such as the house mouse ( *Mus musculus* ), demonstrate obvious nocturnal activity when facing extreme energy demands, such as prolonged cold or hunger (Van Der Vinne et al. 2015).

Some studies have shown that nocturnal activities are vital for the survival of diurnal animals. For example, African elephants ( *Loxodonta africana* ) maintain group cohesion through physical contact and affiliative behaviors at night, thereby regulating social relationships among individuals (Wilson et al. 2006). Research on West African chimpanzees ( *Pan troglodytes* ) highlighted the presence of nocturnal activities, such as foraging behavior, especially in the dry season when high daytime temperatures restrict daytime activity and drive chimpanzees to feed at night as a form of behavioral thermoregulation (Pruetz 2018). These findings suggest that diurnal animals might have different strategies for adapting to environmental challenges while ensuring survival.

Nocturnal vocalization serves as an important adaptive strategy for diurnal animals, helping them evade predators and gain advantages in social or resource competition. For example, some mammals such as black howler monkeys ( *Alouatta pigra* ) use nighttime calls for territorial defense and group coordination (Santini et al. 2015), while African elephants ( *Loxodonta africana* ) produce nocturnal calls that strengthen mother–infant bonds (Stoeger et al. 2011). Similarly, diurnal birds, such as field sparrows ( *Spizella pusilla* ), vocalize at night during the breeding season to attract mates and deter rivals (Celis‐Murillo et al. 2016). According to signal theory, vocalizations act as social tools that convey information about dominance, mating, and kinship, allowing individuals to maintain social cohesion and communication even under limited visibility (Laidre and Johnstone 2013). In complex environments, vocal signals overcome the limitations of vision, enhancing group cohesion and maintaining social structure.

Nocturnal vocal communication in non‐human primates is influenced by a range of ecological and physiological factors. For example, Bearder et al. (2006) demonstrated that nocturnal primates possess visual adaptations specialized for low‐light conditions. By contrast, diurnal species' visual systems are adapted to daytime, face perceptual limitations in conditions with limited light; this might be the reason why they usually rely on acoustic signals at night. Although research on nocturnal vocalization in diurnal primates is limited, Pruetz (2018) recorded the nocturnal activities of chimpanzees in Senegal during the dry season, observing increased social interactions and long‐distance vocal exchanges on brighter, moonlit nights. Although studies on nocturnal vocalization in diurnal primates rarely address individual attributes such as social rank or age, research on their daytime vocal behavior provides valuable insights for understanding it. For instance, Ey et al. (2007) investigated differences in vocal characteristics associated with age and sex in chacma baboons ( *Papio ursinus* ). They reveal that adult males emitted longer calls with lower fundamental frequencies compared to females, particularly after puberty (Ey et al. 2007). Findings from daytime studies suggest that individual attributes, including age and sex, exert measurable effects on vocal production and may similarly influence nocturnal vocal behavior under certain ecological or social contexts (Bouchet et al. 2012; Fan et al. 2018). These findings indicate that nocturnal activities are closely related to ecological conditions, with nocturnal vocal communication serving as an effective social tool for adapting to the environment, coordinating group dynamics, and being influenced by ecological factors, such as temperature, light conditions, food availability, as well as individual attributes like age and social status. These ecological pressures can constrain daytime activity and thereby increase the importance of nocturnal vocal communication as a means of maintaining social cohesion and coordinating group movements.

However, studying nocturnal vocal communication in non‐human primates is particularly challenging, as these behaviors are often difficult to collect. Traditional behavioral observation methods, to some extent, are inadequate for capturing wild animals' behavior in the complex environment, both during the day and at night, due to limitations such as ambient noise, weather fluctuations, and the range of monitoring equipment. For example, in a nocturnal activity study of proboscis monkeys ( *Nasalis larvatus* ), GPS collars were used, but the inability to perform individual identification was a limitation of that method (Kooros et al. 2022). Similarly, the difficulty of individual recognition is a common challenge for passive acoustic monitoring (PAM), as acoustic data are typically collected at the group rather than the individual level. Nevertheless, increasing research highlights that PAM and infrared surveillance technologies are essential tools to overcome such challenges, particularly under nocturnal conditions. PAM provides efficient sound data through long‐term, automatic recording devices, reducing human interference and creating new possibilities for studying wildlife behavior and ecology (Moreira Sugai et al. 2019; Zwerts et al. 2021). For example, among wild chimpanzees, passive acoustic arrays successfully located loud calls, revealing patterns of behavioral and social interactions and demonstrating significant influences of environmental variables, such as wind speed and temperature, on sound propagation (Crunchant et al. 2022). The Acoustic Complexity Index (ACI), which quantifies the variability in biotic sounds within a recording, has also been identified as a valuable tool in this context (Farina et al. 2011). By measuring acoustic complexity, the ACI has often been applied to assess soundscape diversity. However, in contexts where a focal species dominates the acoustic environment, ACI can also serve as a proxy for the vocal activity of that species (Pieretti 2011; Bradfer‐Lawrence et al. 2019). Similar index‐based or automated passive acoustic approaches have been successfully applied in primate research to quantify calling activity or diel patterns when direct behavioral observations were limited, for example in studies using passive acoustic monitoring to detect and monitor African great apes and forest guenons (Heinicke et al. 2015; Kalan et al. 2016) and to describe diel vocal behavior in howler monkeys (Do Nascimento et al. 2021). These studies demonstrate that the ACI can be effectively applied to investigate temporal patterns of vocal activity in contexts where a single species dominates the acoustic environment. Similarly, infrared camera traps have revealed unexpected nocturnal activity patterns in primates through video data, such as in a study of the Guizhou snub‐nosed monkey *Rhinopithecus brelichi* , where individuals exhibited nocturnal activity beyond twilight periods, highlighting their behavioral flexibility in adapting to ecological conditions (Tan et al. 2013). However, few studies include both infrared video and passive sound technology. For example, Gazagne et al. (2023) documented the application of thermal infrared imaging together with passive acoustic monitoring in primates. This is because infrared surveillance technology allows researchers to capture animal behavior data in low‐light environments; combining this technique with passive acoustic monitoring can be essential for exploring nocturnally active primate species (Pruetz 2018).

Tibetan macaques ( *Macaca thibetana* ), a diurnal primate species endemic to China (Ogawa and Takahashi 2003), served as the focal species of this study. Although previous studies have classified their vocal repertoires during daytime activities (Bernstein et al. 2016), research on their nocturnal vocalization remains limited. Previous studies have documented the vocal repertoire of Tibetan macaques in detail, describing specific call types and their associated behavioral contexts (Bernstein et al. 2016). Therefore, the present study does not aim to redefine their vocal repertoire, but rather focuses on the nocturnal occurrence patterns of these vocalizations and their behavioral associations. Spectrograms shown in this study are included solely to illustrate representative examples of vocalizations and aid visual interpretation, not for acoustic classification or additional signal analysis. Additionally, Tibetan macaques live in multi‐male, multi‐female groups characterized by a linear dominance hierarchy and strong matrilineal kinship. Their social structure relies on coordination and consensus, with social centrality influencing group dynamics (Wang et al. 2015). Moreover, their stable social structure, clear dominance hierarchy, specific nocturnal activity ranges, and generally reliable yet occasionally limited individual identification make them an ideal model species for decoding nocturnal vocal behaviors through the integration of PAM and infrared surveillance technologies.

This study explored the process of nocturnal vocalization and its social functions among Tibetan macaques. Based on previous research, we hypothesized that: (1) Nocturnal vocalizations may occur in Tibetan macaques, and if present, they would exhibit temporal variation in frequency, reflecting potential patterns of nocturnal vocal activity, as diel variations in activity rhythms and vocal behaviors have been reported in other animals (Kronfeld‐Schor and Dayan 2003; Pruetz 2018). (2) Nocturnal vocalization count would vary significantly with ecological factors, as well as sex, age, and social relationships, since ecological pressures such as temperature and predation risk, and individual attributes like age and dominance, have been shown to influence primate sounds (Bearder et al. 2006; Bouchet et al. 2012; Fan et al. 2018). (3) Potential distinct peaks in nocturnal vocalizations would be associated with different social contexts, such as competition‐related vocalizations in the evening and social coordination in the late night, deriving from evidence that vocal signals often serve context‐dependent functions in maintaining cohesion, regulating competition, and facilitating group coordination (Mitani and Brandt 1994; Wilson et al. 2006).

## 2 Materials and Methods

### 2.1 Study Subjects and Location

**FIGURE 1:**
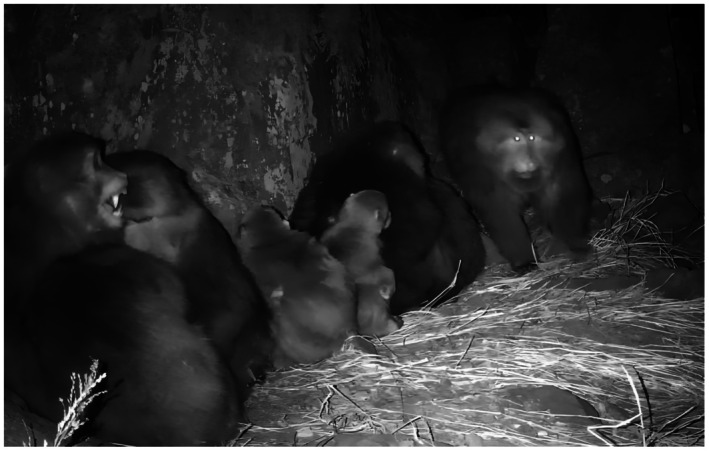
A Tibetan macaque ( *Macaca thibetana* ) photographed at night using an infrared camera. The image illustrates the typical appearance of the species and the environmental context in which nocturnal observations were conducted.

This study was conducted from July 2023 to May 2024 in Huangshan, Anhui Province, China (30°04′25.1′′ N, 118°08′59.3′′ E). The study subjects were wild Tibetan macaques ( *Macaca thibetana* ) inhabiting the Huangshan Monkey Valley, which is part of the Huangshan Scenic Area, a nationally protected reserve. The study group (YA1 group) included 10 adult females (≥ 5 years), 9 adult males (≥ 7 years), 6 subadults (4–6 years), 8 juvenile females (0–3 years), and 16 juvenile males (0–4 years). Due to the challenges of identifying infants and the validity of data at night, the analysis focused on the individuals consisting of 10 adult females, 9 adult males, and 5 subadult and juvenile monkeys. The YA1 group has been continuously monitored since 1986, and the individuals are habituated to the presence of observers, which facilitates individual identification using night‐vision 4G cameras equipped with advanced technology (Figure 1).

### 2.2 Behavioral Observation

Observations were conducted daily from 08:30 to 17:30, with 6–8 h per day, 6–7 days a week. We used the focal animal sampling method (Altmann 1974) during each 15‐min observation interval to record the behaviors of individuals in the group (*N* = 19). Behaviors were recorded based on predefined categories (Li 1999) and classified into four major types: aggression‐related behaviors (e.g., agonistic support), social‐related behaviors (e.g., bridging), sleep‐related behaviors (e.g., huddling sleep), and other behaviors (e.g., appearing in the field of view). A detailed description of each behavior was provided in Table 1. The goal of this data collection was to calculate the social centrality of the participating individuals. In addition, instances of aggression and submission were recorded using an ad libitum sampling method.

**TABLE 1:** Behavioral categories and definitions.

Behavior type	Behavior	Definition
Aggression‐related behaviors	Agonistic support	Refers to the behavior of an individual in which a participant in an ongoing struggle to support one party and attack the other
	Ground slap	One male individual supports with one hand on the ground, the other flaps the ground, and stares to the other male individual, then it looks down
	Long lunge	An individual directs a lunge > 2 body lengths to another individual but does not go into a full chase
	Short lunge	Short lunge: An individual directs a lunge < 2 body lengths to another individual
	Redirection	When A is attacked by B, A responds immediately by attacking C. C is an individual that is nearby and lower‐ranking than both A and B. In some cases, B will join the attack against C
	Stare	An individual looks directly at another individual with its eyes wide open and with its shoulders raised for about 3–5 s. The staring individual appears as if it is preparing to lunge or chase the recipient of the stare
Social‐related behaviors	Bridging	Bridging involves three individuals, an infant or young juvenile and (a) two adult males, (b) one adult male and one subadult male, or (c) two adult females. The two older individuals hold the infant on its back, lower their heads and lick the belly and/or genitals of the infant. They will often teeth‐chatter and vocalize excitedly. The infant is usually male and his penis becomes erect immediately. Other infants, particularly females will approach a bridging triad with excitement as if they wish to participate
	Embrace	Two individuals hold each other while face to face. Each partner will reach with one hand and attempt to touch the genitals of the other. Both partners typically teeth‐chatter and vocalize excitedly
	Holding an infant	An adult male holds an infant and may carry it ventrally. Usually the infant is male. This apparently serves to invite other adult males to engage in social interaction with the adult male
	Homosexual mount	One male (usually the lower‐ranking) grabs the back hair of another male and mount from behind, using the full ankle‐clasp posture. Both males teeth‐chatter and scream excitedly. Then the mounter dismounts. The duration of the mount is about 3–5 s. This may be a simple friendly gesture or a post conflict behavior
	Proximity < 1 m	Two or more individuals are sitting or lying within 1 m of one another
	Penis showing	This is a ritualized behavior shown by juvenile males toward adult males. Usually, the lower‐ranking male approaches the higher‐ranking male, raises one leg and displays his penis. The higher‐ranking male puts his head on the belly of the lower‐ranking male and licks or touches the penis with his hand. The juvenile may show his penis from a reclining position
	Penis sucking	A young male approaches an adult male and jumps on his head. The adult male holds the younger male by the waist in such a way that his mouth can reach the young male's penis. The adult sucks the young male's penis and then the younger male leaves
Sleep‐related behaviors	Huddling sleep	A group of two or more individuals sleeping in close physical contact
	Sleep	An individual enters a sleeping state alone
	Sleeping with eyes closed	Eyes are closed, and the individual enters a sleeping state, either alone or in a group
	Wake up with eyes open	While in a sleeping state, the individual wakes up and opens its eyes
	Raise the head	While in a sleeping state, the individual wakes up due to external stimuli and lifts its head
Other behaviors	Appear	An individual enters the field of view of the camera
	Self‐scratch	Movement of the hand or foot during which the fingertips are drawn across the fur or skin
	Sit	Individuals use their hips against the ground to support their weight

### 2.3 Recordings of Nocturnal Vocalizations and Related Behaviors

To capture nocturnal activities, we used DS‐2XS2T46XM infrared cameras (Hikvision, Hangzhou, China) with 4G night‐vision capabilities, providing high‐resolution footage in low‐light conditions. These cameras were installed on trees along the cliff edge, where Tibetan macaques frequently rest on small, relatively flat rock platforms. The camera locations were strategically chosen to cover both the sleeping and active zones, ensuring comprehensive monitoring of group dynamics. The cameras were synchronized with passive acoustic recording devices (Luyin, Qinghai, China; available at https://www.bio‐equip.com/otherproduct88432.html), operating at a sampling rate of 0–48 kHz to capture vocalizations across a broad frequency range. Tibetan macaque vocalizations typically fall within the range of approximately 0–24 kHz (Bernstein et al. 2016). Thus, setting the sampling rate at 48 kHz ensured that the full range of species‐specific vocalizations could be captured with sufficient resolution. The recorders were fixed at approximately 1.5 m above the ground, and multiple infrared cameras were distributed around each recorder to monitor the entire group, ensuring that acoustic data could be cross‐referenced with visual observations of individual behaviors. This setup enabled group‐level monitoring of macaque behavior and vocalizations, with GPS time‐stamping ensuring temporal alignment of video and audio data. To identify the habitual activity zones, we conducted a survey of their sleeping sites, confirmed activity hotspots through footprints and nocturnal observations, and installed 25 cameras in these areas. In addition to the cameras, we also deployed two passive acoustic recording devices to continuously monitor vocal activity. During data collection, we reviewed the nocturnal videos and used the all‐occurrence sampling method to document individual behaviors, accurately identifying each macaque based on distinctive morphological features observed during the daytime, such as scars, fur color, body size, and facial expressions.

In this study, nocturnal vocalization and its related behaviors were defined as any activities occurring within 10 s before or after an individual vocalizing were recorded. Specifically, behaviors occurring within 10 s before the first vocalization and 10 s after the last vocalization were documented. Vocalizations separated by intervals of less than 5 s were considered part of the previous vocalization event and processed as the same entry. Additionally, behaviors of surrounding individuals within the 10‐s period were simultaneously recorded to provide background information for further analysis of the social interactions and environmental context of the vocalization behavior.

The 10‐s behavioral window around vocalizations was selected for several reasons. First, primate vocalizations are typically immediate social or behavioral signals, and a 10‐s window effectively captures behaviors directly linked to vocalizations, while excluding unrelated behaviors that may occur over longer periods. Second, a brief time window (e.g., 10 s) is both scientifically and practically suitable for analyzing primate vocalization and behavioral interactions (Notman and Rendall 2005). Moreover, the accuracy and synchronization limitations of nocturnal monitoring equipment require a shorter time frame, and the 10‐s window helps ensure the accuracy and reliability of the analysis. The behavioral context within this 10‐s window was classified into social, aggression, sleep, and other behaviors (Table 1).

### 2.4 Video and Audio Analysis

Across the study period, we conducted 1218 h (73,080 min) of videos and recorded 1768 high‐quality vocalization events. Following preliminary screening, 442 high‐quality vocalization records with corresponding behavioral contexts were selected for analysis. These vocalizations were obtained from passive acoustic recorders and subsequently verified using synchronized infrared video recordings to ensure accurate attribution to Tibetan macaques. The final selected time period was from November 2023 to January 2024, ensuring all data were valid nocturnal data based on astronomical twilight times.

While recording vocalization behaviors, we also documented the identity of the vocalizing individual, including their sex, age, and kinship based on long‐term monitoring of the group. Kinship was quantified by the number of direct relatives each individual had within the group. Social rank was determined using DS (David's Score) values (Gammell et al. 2003), with binary antagonistic interactions indicating dominance relationships between individuals (Li 1999). Higher DS values corresponded to higher social rank, which was categorized into high, medium, and low levels using K‐means clustering. Analyses were conducted in R version 4.3.6.

To calculate individual social centrality, we employed the dyadic association index (DAI) to assess interactions between individuals A and B:

$$\mathrm{DAI}=D_{\mathrm{ab}}/\left(D_{a}+D_{b}-D_{\mathrm{ab}}\right)$$

where *D*_ab_ represents the total time individuals A and B spend within 1 m of each other, and *D*_a_ and *D*_b_ represent their respective focal sampling times (Bracken et al. 2022). Using the DAI matrix, we calculated the eigenvector centrality coefficient in R version 4.3.6. A high eigenvector centrality coefficient indicates that an individual is highly connected to many others, especially to those with high centrality (Whitehead 2008).

At the same time, passive acoustic monitoring equipment was used to record the nocturnal vocalization data of Tibetan macaques, covering the period from 17:00 to 06:00. Vocal activity levels were quantified via the ACI (Pieretti 2011):

$$\mathrm{ACI}=\sum_{i=1}^{N}\left(\sum_{f=f_{\min}}^{f_{\max}}\frac{\left|P\left(f,t_{i}\right)-P\left(f,t_{i-1}\right)\right|}{P\left(f,t_{i}\right)+P\left(f,t_{i-1}\right)}\right)$$

In this study, we calculated the ACI to quantify variations in sound across different frequencies and time intervals. Audio files were divided into 600‐s segments, referred to as time windows (*t*_*i*_). For each time window, we extracted the power spectral density (*P*) of different frequency components (*f*), represented as *P* (*f*, *t*_*i*_). By calculating the intensity change at the same frequency between adjacent time windows “|*P*(*f*, *t*_*i*_) − *P*(*f*, *t*_*i*_ − 1)|” and normalizing with “*P*(*f*, *t*_*i*_) + *P*(*f*, *t*_*i*_ − 1)”, we obtained the ACI value for each time window and frequency. The ACI values for all frequency components within each time window, and the results from all time windows were aggregated to compute the total ACI value for the entire audio file. This method effectively captures subtle variations in the sound signal over time and frequency, making it suitable for analyzing the activity level of biological vocalizations while filtering out constant noise, ensuring more accurate results.

Although the ACI is commonly used to measure overall soundscape complexity, it has also been employed to estimate the vocal output of dominant species in acoustically simple environments (Pieretti 2011). In our study setting, nocturnal acoustic recordings were overwhelmingly dominated by Tibetan macaque vocalizations, with minimal contributions from other biological or environmental sound sources. To validate the suitability of ACI as a proxy for macaque vocal activity, we conducted a correlation analysis between ACI values and manually recorded vocalization counts across matched hourly intervals. The results revealed a significant positive correlation (*r* = 0.76, *p* < 0.001), indicating that ACI reliably reflects temporal patterns of macaque vocal activity under our study conditions, while manual counts served as the ground‐truth reference. This validation step reinforces the methodological appropriateness of using ACI for subsequent statistical analyses in this study. To descriptively identify baseline vocal activity and highlight peak or trough hours, we defined the baseline as the median of hourly vocalization counts (Q2), with peaks identified as values above the upper quartile (Q3) and troughs as values below the lower quartile (Q1). This quartile‐based method provides a descriptive overview of variation in activity levels but does not constitute a formal statistical test of bimodality.

Additionally, we obtained daily ecological data from 17:00 to 06:00, including temperature, humidity, precipitation, wind speed, wind direction. These ecological variables provided essential background information to explore the relationship between environmental conditions and vocalization behavior, helping us gain a deeper understanding of the dynamics under varying conditions. All meteorological data used in this study were obtained from the China Meteorological Data Service Center (http://data.cma.cn/), the official national platform for standardized climate and weather information in China.

### 2.5 Statistical Analysis

#### 2.5.1 Effect of Individual Attributes on Vocalization Count

To investigate the effects of individual attributes on vocalization count, we employed a generalized linear mixed model (GLMM) (McCullagh 2019). The model was built using the lme4 package in R (Bates et al. 2015), with vocalization count as the response variable and individual attributes (rank, sex, age, social vector centrality, and kinship) as fixed effects. Individual ID was incorporated as a random effect to account for individual heterogeneity. Given that vocalization count represents count data, we employed a Poisson distribution for model fitting (McCullagh 2019). To assess multicollinearity among the independent variables, we calculated the variance inflation factor (VIF) (Gray 1998). All VIF values were below 5 (Table S1), which is commonly considered the threshold indicating no multicollinearity. The results of the model were organized and summarized with the broom.mixed package (Bolker and Robinson 2018).

#### 2.5.2 Effect of Environmental Factors on ACI

In this study, we quantified vocalization behavior and acoustic activity levels using both vocalization count and ACI. To ensure accuracy in data analysis, we first assessed the normality of the data using the Shapiro–Wilk test (Shapiro and Wilk 1965). If the data did not follow a normal distribution, we examined the relationship between vocalization count and ACI values using Spearman correlation (Spearman 1961). To investigate the impact of environmental factors on vocalization behavior, we used a generalized additive model (GAM) (Wood 2011) to examine the relationship between the ACI and environmental factors, including temperature, humidity, precipitation, wind speed, and wind direction. To ensure the validity of the model, we first assessed the collinearity among these environmental variables. In addition to temperature, humidity, precipitation, and overall wind direction, we also included zonal wind (east–west), meridional wind (north–south), surface wind speed, and ground‐level solar radiation in our analysis. These variables were included because they represent key ecological components that may influence acoustic transmission and vocal behavior under nocturnal conditions. Specifically, zonal wind refers to the east–west component of horizontal wind, and meridional wind represents the north–south component. Distinguishing between these wind directions was ecologically relevant because the macaque sleeping cliff faces west‐to‐east, and wind direction could therefore influence the propagation of sound across the valley. Surface wind speed reflects the total near‐ground airflow intensity, while surface horizontal radiation was incorporated as an exploratory proxy for residual nocturnal brightness and thermal variation. Although direct studies linking this variable to primate vocal activity are lacking, previous research has shown that nocturnal light conditions—such as moonlight or reflected radiation—can affect animal activity rhythms and acoustic communication (Starr et al. 2012; Kronfeld‐Schor et al. 2013; Dickerson et al. 2023). Including this variable thus allowed us to consider potential indirect effects of night‐time luminous and thermal variation on acoustic detectability. Variance inflation factor (VIF) analysis confirmed that these variables were not highly collinear, allowing their inclusion in the model without multicollinearity concerns (Table S2). Four candidate GAM models (with and without interaction terms) were fitted using the mgcv package in R (Wood 2011, 2017). Non‐significant predictors were stepwise removed, and the final model retained only significant environmental variables and interactions, which were then used to identify ecological factors associated with ACI. The model results included parameter estimates and significance tests of smooth terms, while visualization tools were used to depict univariate and interaction effects of the key variables. Additionally, we used the Friedman test to compare the frequency of different behavior types around vocalizations. All analyses were performed in R version 4.3.6, with a significance level set at 0.05.

### 2.6 Ethical Statement

This study was conducted in collaboration with the Huangshan Monkey Management Center and the Huangshan Forestry Bureau of China. It was entirely observational, involving no invasive experiment on wild primates and ensuring no impact on the welfare of Tibetan macaques. Thus, no review from an institutional ethics committee in China was required. The research complies with the ethical guidelines set by the China Wildlife Conservation Association and the Wildlife Protection Law of the People's Republic of China.

## 3 Results

### 3.1 Analysis of Nocturnal Vocalization Patterns

**FIGURE 2:**
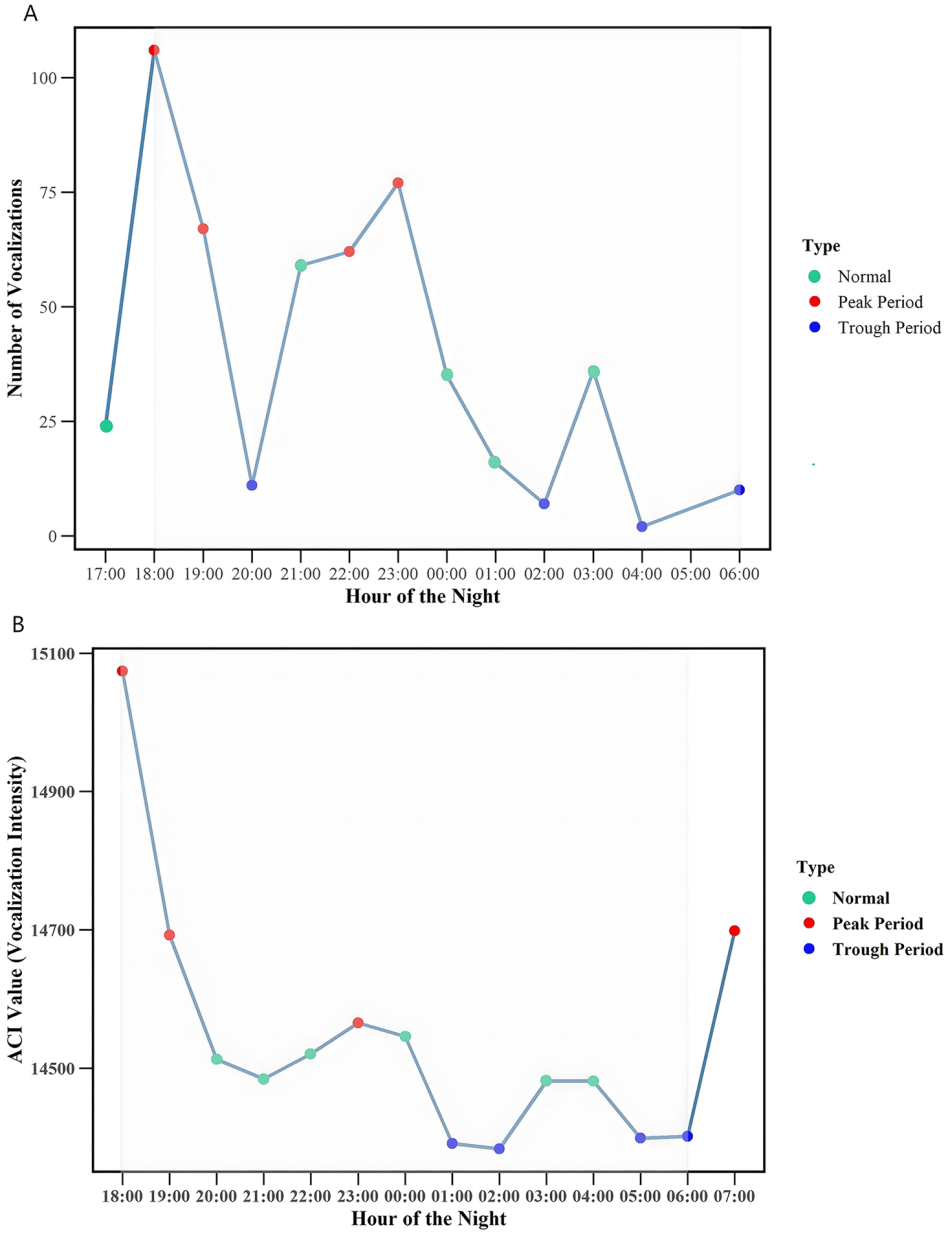
Temporal variation in nocturnal vocal activity and acoustic complexity in Tibetan macaques. (A) Hourly variation in nocturnal vocalization counts. The line represents the total number of vocalizations recorded per hour between 17:00 and 06:00, with points colored to indicate periods classified as peak (red; above Q3), normal (green; between Q1 and Q3), and trough (blue; below Q1) activity levels. A descriptive bimodal trend is observed, with elevated vocal activity around 18:00–19:00 and again between 21:00 and 23:00. Shaded areas denote the main nighttime monitoring period. (B) Temporal variation in Acoustic Complexity Index (ACI). The line shows hourly ACI values between 18:00 and 07:00, with points classified as peak (red; above Q3), normal (green; between Q1 and Q3), and trough (blue; below Q1). ACI values exhibit higher levels around 18:00–19:00 and again near 23:00, though the timing and magnitude of peaks differ from those of vocalization counts. Shaded areas denote the main nighttime monitoring period.

Based on 442 recorded vocalization events, the distribution of vocalization count between 17:00 and 06:00 was analyzed (Figure 2A). A possible bimodal trend was observed, with distinct peaks at 18:00–19:00 and 21:00–23:00, marking these as the most active periods of vocal activity. Vocalization count gradually decreased as the night progressed, reaching its lowest point between 02:00 and 04:00. Although some vocalizations could be assigned to specific individuals, the limited number of individual‐level records precluded robust modeling; therefore, group‐level totals were used to characterize nocturnal vocalization patterns. To statistically test this bimodal trend, we applied a GAM with hour of night as a smooth term, which confirmed a significant non‐linear effect of time on vocalization count (df = 6.87, *F* = 4.92, *p* < 0.001), supporting the visual observation of a possible bimodal distribution.

To better understand the level of nocturnal acoustic activity, we calculated ACI values for each time period (Figure 2B). Although ACI values exhibited a broadly similar trend to vocalization counts, showing higher activity during the early evening and a subsequent decline toward midnight, the timing and magnitude of peaks differed between the two measures. ACI values peaked at 22:00–23:00 which exhibited only minor fluctuations beyond this period. This discrepancy suggests that vocalization count and acoustic complexity are likely influenced by distinct behavioral or environmental factors. The results revealed a significant positive relationship between vocalization count and ACI (Spearman correlation coefficient *r* = 0.79, *p* = 0.0036). This finding supports the hypothesis of a possible bimodal vocalization pattern and underscores the complex interplay between nocturnal vocalization behavior and vocal activity level in Tibetan macaques.

### 3.2 Influence of Socio‐Biological Factors on Vocalization

A generalized linear mixed model (GLMM) was used to analyze the effects of socio‐biological factors on vocalization count. Results from the GLMM analysis (Table 2) indicated that sex, age, social centrality, and kinship significantly influenced vocalization count. Specifically, females vocalized less frequently than males (*z* = −4.671, *p* < 0.001), and age showed a negative correlation with vocalization count (*z* = −2.932, *p* < 0.01). Individuals with higher social centrality exhibited lower vocalization count (*z* = −3.274, *p* < 0.01), whereas increased kinship showed a positive correlation with vocalization count (*z* = 3.013, *p* < 0.01). Rank, however, did not significantly influence vocalization count (*z* = −1.274, *p* = 0.202).

**TABLE 2:** Effects of predictor variables on Tibetan macaque vocalization count in the GLMM analysis.

Predictor variable	Estimate	SE	*z* value	*p*
Intercept	7.748	1.306	5.931	< 0.001
Rank	−0.285	0.224	−1.274	0.202
Sex	−1.909	0.409	−4.671	< 0.001
Age	−1.342	0.458	−2.932	< 0.01
Centrality	−2.163	0.661	−3.274	< 0.01
Relatives	0.249	0.083	3.013	< 0.01

### 3.3 Influence of Ecological Factors on Vocalization

**FIGURE 3:**
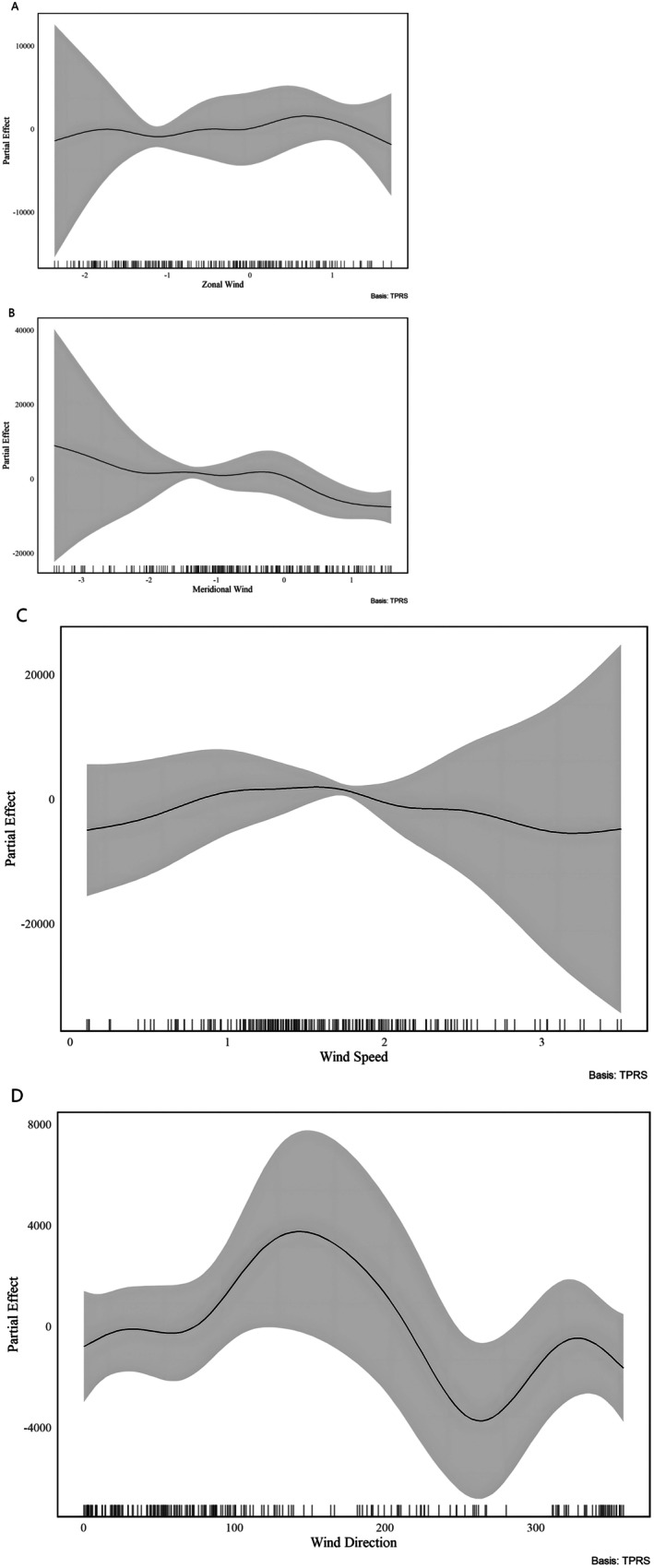
Partial effects of ecological variables on the Acoustic Complexity Index (ACI) from the GAM analysis. (A) Partial effect of zonal wind (east–west component) on ACI. The solid line represents the estimated smooth term, and the shaded area indicates the 95% confidence interval. Tick marks along the *x*‐axis indicate data density. (B) Partial effect of meridional wind (north–south component) on ACI, showing a nonlinear relationship across the observed range. (C) Partial effect of surface wind speed on ACI. Higher wind speeds show an associated decline in ACI estimates toward the upper range, though confidence intervals widen at extreme values. (D) Partial effect of wind direction (0°–360°) on ACI. The curve illustrates periodic variation in ACI estimates across different incoming wind orientations. All smooth terms were fitted using thin‐plate regression splines (TPRS). Shaded gray bands represent 95% confidence intervals.

**FIGURE 4:**
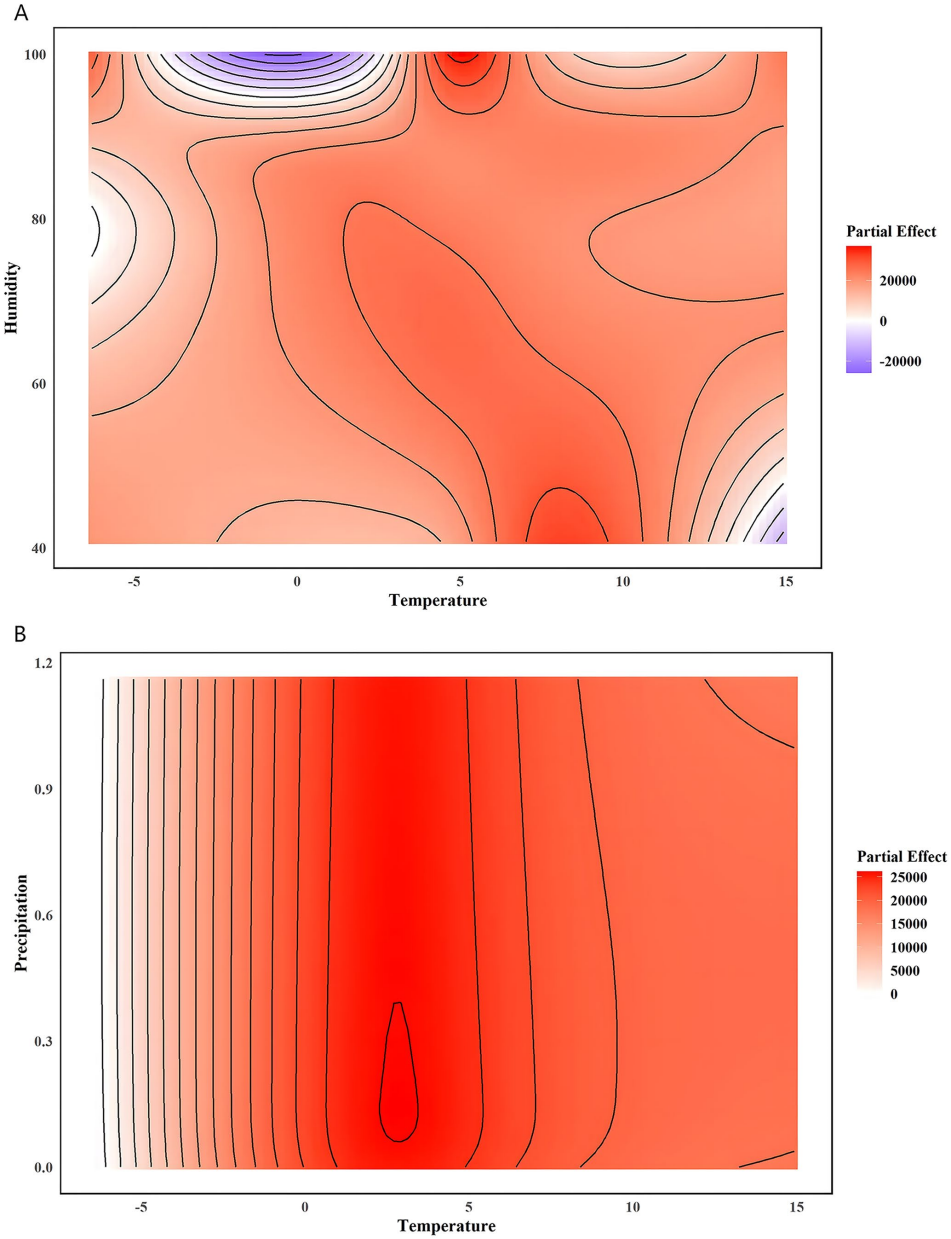
Interaction effects of climatic variables on the Acoustic Complexity Index (ACI). (A) Interaction effect of temperature and precipitation on ACI. This contour plot illustrates how combinations of temperature and precipitation influence acoustic complexity. Warmer temperatures combined with moderate precipitation show elevated ACI values, whereas extremely low or high values of either variable correspond to lower ACI levels. Color gradients indicate the magnitude of partial effects, and contour lines highlight zones of rapid change. (B) Interaction effect of temperature and humidity on ACI. This panel shows how ACI varies across temperature–humidity combinations. Higher ACI values occur under conditions of low temperature and high humidity, while decreases are observed at high temperature with low humidity. Warmer colors represent stronger positive partial effects, and cooler colors indicate negative effects.

The GAM model achieved an adjusted *R* ^2^ of 0.866, explained over 86% of the variance in ACI. Results indicated that meridional wind (df = 6.925, *F* = 2.176, *p* < 0.05), zonal wind (df = 6.223, *F* = 3.254, *p* < 0.01), wind direction (df = 6.155, *F* = 2.15, *p* < 0.05), and surface wind speed (df = 4.483, *F* = 8.276, *p* < 0.001) had significant effects on ACI values (Table 3). As meridional and zonal winds increased, ACI values initially rose and then declined (Figure 3A,B). In contrast, ACI values increased significantly with rising surface wind speed (Figure 3C). Wind direction, ranging from 0° to 360° (representing winds from north through east, south, and west), showed a periodic influence on ACI. Among all directions, easterly winds (90°) and westerly winds (270°) had the strongest impact on ACI (Figure 3D). This pattern may be related to the orientation of the sleeping cliff, which faces south. Additionally, a significant interaction between temperature and humidity was detected (df = 20.447, *F* = 7.874, *p* < 0.001). ACI values increased under conditions of low temperature and high humidity, whereas high temperature combined with low humidity reduced vocal activity. A significant interaction was also observed between temperature and precipitation (df = 7.743, *F* = 1.012, *p* < 0.01), with ACI values increasing under conditions of low temperature and high precipitation (Figure 4).

**TABLE 3:** Effects of environmental factors on the Acoustic Complexity Index (ACI) of Tibetan macaques in the GAM analysis.

Term	df (Ref)	*F* value	*p*
Temperature	3.375 (4.252)	0.031	1
Humidity	4.939 (5.7)	0.149	0.982
Precipitation	2.412 (2.914)	1.266	0.213
Zonal wind	6.925 (7.913)	2.176	< 0.05
Meridional wind	6.223 (7.182)	3.254	< 0.01
Surface wind speed	4.483 (5.471)	8.276	< 0.001
Wind direction	6.155 (7.229)	2.15	< 0.05
Surface horizontal radiation	1 (1)	0.045	0.832
Temperature:humidity	20.447 (20.637)	7.874	< 0.001
Temperature: precipitation	7.743 (19)	1.012	< 0.01

### 3.4 Social Functions of Nocturnal Vocalization

**FIGURE 5:**
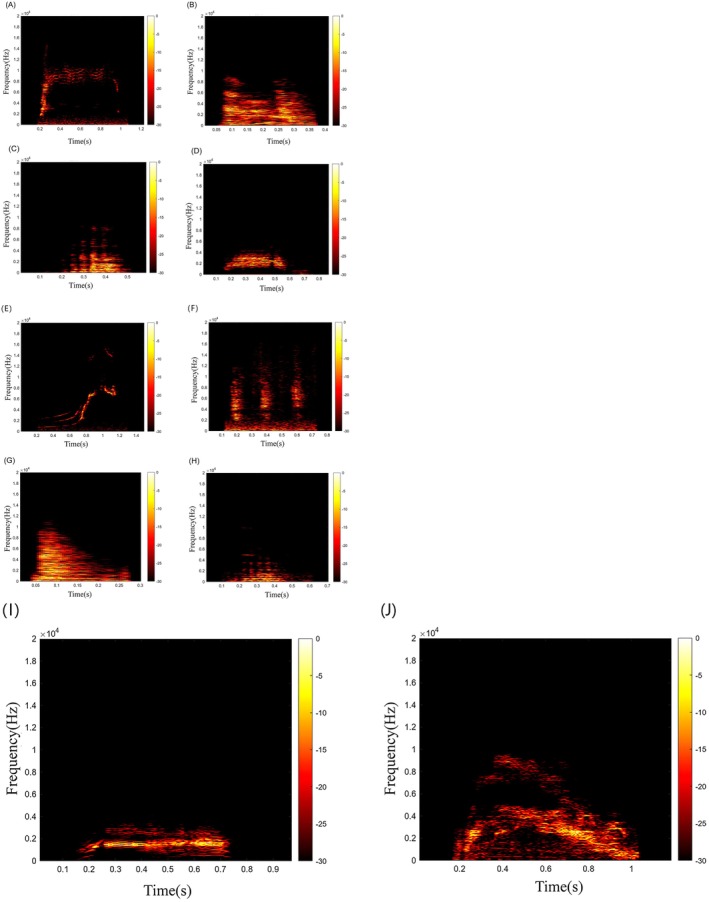
Spectrograms of Tibetan macaque vocalizations recorded during aggression‐related and social‐related behavioral contexts. (A–D) Show representative spectrograms of vocalizations associated with aggression‐related behaviors. (E–J) Show representative spectrograms of vocalizations associated with social‐related behaviors. Color scales indicate relative amplitude (dB), with warmer colors representing higher energy.

**FIGURE 6:**
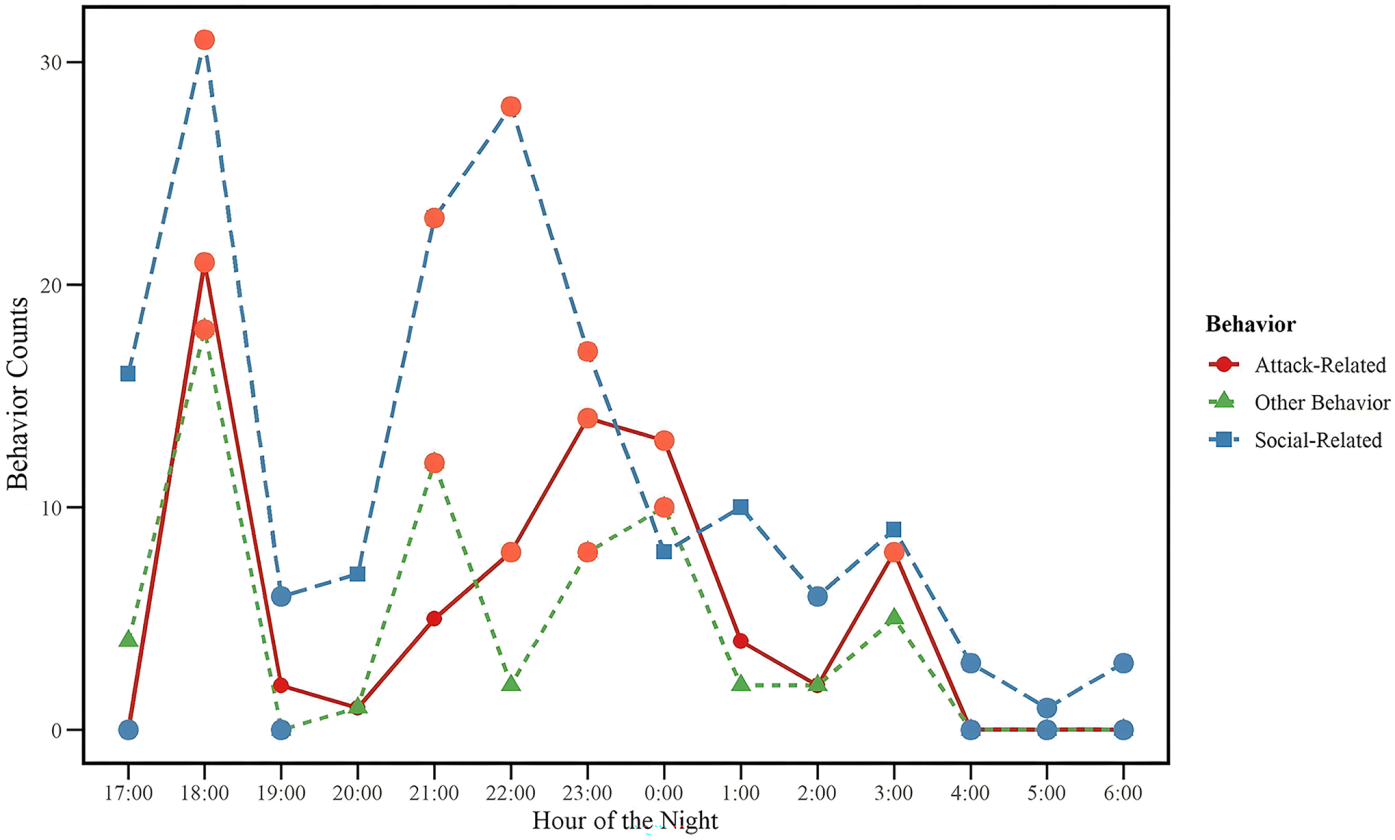
Temporal rhythm of nocturnal behaviors in Tibetan macaques (17:00–06:00). Hourly variation in the occurrence of aggression‐related behaviors (red circles and solid line), social‐related behaviors (blue squares and dashed line), and other behaviors (green triangles and dotted line). The figure illustrates how different behavior types fluctuate across the nocturnal period, with social‐related behaviors showing particularly elevated activity around 18:00 and 22:00.

This study investigated the nocturnal behavioral rhythms of Tibetan macaques, with a specific focus on vocalization and its behavioral contexts. Behaviors occurring within 10 s before and after vocalizations were recorded and categorized as social‐related, aggression‐related, sleep‐related, or other behaviors, with the distributions of their counts analyzed (Figure 5). The results indicated that around 18:00, diverse behaviors were frequently observed, potentially linked to competition for sleeping spots. Between 21:00 and 22:00, social‐related behaviors occurred at significantly higher counts compared to other behaviors, suggesting heightened social interaction during this period. The Friedman test results revealed significant differences in the nocturnal distribution of counts for social‐related, aggression‐related, and other behaviors (*p* < 0.05) (Figure 6).

## 4 Discussion

The findings of this study largely corroborate the proposed hypotheses. First, consistent with hypothesis 1, the nocturnal vocalizations of Tibetan macaques displayed a possible bimodal trend, with two peaks occurring in the early evening (18:00–19:00) and late night (22:00–23:00). These peaks suggest that vocalizations at different times likely serve distinct behavioral functions. Second, partially supporting hypothesis 2, we found that individuals with lower social centrality vocalized more frequently, suggesting that vocalizations may function as a strategy for enhancing social integration within the group. However, contrary to expectations, high‐ranking individuals did not vocalize more frequently, implying that the factors driving vocalization behavior in Tibetan macaques may be more complex than initially assumed. Additionally, the positive correlation between vocalization count and kinship bonds further highlights the role of vocalizations in maintaining social ties within the group. Regarding ecological conditions, hypothesis 2 was also partially supported, as environmental factors such as wind speed, temperature, and humidity significantly influenced the ACI. Although this study did not directly compare vocal behaviors between day and night, we observed that the types of behaviors associated with vocalizations at night were consistent with those commonly seen during the daytime, and these behaviors served as important contextual backgrounds for nocturnal vocalizations. This suggests that environmental conditions, such as wind speed and humidity, may shape the acoustic patterns detected in our recordings. While these associations imply that ecological factors could influence the efficiency of vocal communication, it remains to be tested whether Tibetan macaques actively modify their vocalization strategies in response to such conditions. Finally, hypothesis 3 was supported by our findings, with evening vocalizations associated with competitive behaviors (e.g., competition for sleeping sites) and late‐night vocalizations primarily serving social functions, such as group coordination. This suggests that nocturnal vocalizations in Tibetan macaques reflect biological rhythms but also play a crucial role in maintaining social structure and adapting to environmental challenges. Overall, these results provide valuable insights into the nocturnal behavior of Tibetan macaques and contribute to a broader understanding of acoustic communication in primates, particularly regarding social dynamics and environmental adaptation.

The possible bimodal trend observed in Tibetan macaques reflects both their social behaviors and ecological adaptations. The evening peak (18:00–19:00) is likely linked to pre‐sleep social interactions and competition for optimal sleeping sites. Similar dusk‐related peaks have been documented in other primates, such as the black and gold howler monkeys ( *Alouatta caraya* ), where vocalizations help maintain group cohesion and minimize conflict before settling for the night (Perez‐Granados and Schuchmann 2021). In contrast, the late‐night peak (22:00–23:00) observed in Tibetan macaques may reflect periods of fragmented sleep and intermittent wakefulness, which appear to be a species‐specific nocturnal feature rather than a pattern shared with howlers. Recent field research using night‐vision camera systems revealed that Tibetan macaques spend approximately 18% of the night in active states, including movement, rest, and social behavior, with a distinct activity peak observed between 23:00 and 01:00 (Yang et al. 2025). This pattern suggests that sleep in this species is not continuous and may allow for low‐level behavioral interactions under specific ecological or social contexts. Comparable behavior has been documented in other diurnal primates. For instance, Guianan red howler monkeys ( *Alouatta macconnelli* )—though considered diurnal—exhibit pronounced vocal peaks around 05:00 a.m., prior to nautical twilight, and are largely silent during the day (Do Nascimento et al. 2021). These howls are typically emitted from roosting sites and are believed to serve functions related to territory defense or group synchronization. These findings collectively support the notion that late‐night vocalizations in diurnal primates may be adaptive rather than aberrant. In Tibetan macaques, such vocal output may serve to maintain social cohesion, enable thermoregulatory huddling coordination, or facilitate spatial reorientation during nocturnal awakenings, especially under low‐light conditions when visual cues are limited.

Sex, age, social centrality, and kinship had a significant influence on the vocalization count of Tibetan macaques. Females vocalized less frequently than males, consistent with previous research suggesting that females in primate groups typically engage more in social functions than in vocal signaling. Aung et al. (2023) reported sex‐related differences in vocalization behavior in golden snub‐nosed monkeys ( *Rhinopithecus roxellana* ), with males in high‐competition groups tending to use low‐frequency calls to attract mates or deter rivals (Aung et al. 2023). Our findings, which show that younger Tibetan macaques vocalized more frequently, suggest that they may use vocalizations to integrate into the group and acquire social skills. This aligns with Coye et al. (2022), who highlighted that young primates in general often learn essential acoustic signals through imitation and frequent social interactions, aiding their adaptation to complex group life. Furthermore, individuals with lower social centrality vocalized more frequently, supporting the hypothesis that peripheral members of primate groups use vocalizations to increase their social presence and maintain relationships. For example, Cheney and Seyfarth (2018), working on baboons (*Papio* spp.), argued that individuals in social networks often rely on vocal signals to reduce uncertainty and enhance their presence in competitive or cooperative contexts. Finally, the positive correlation we observed between kinship and vocalization count suggests that vocalizations play a key role in reinforcing kinship bonds, consistent with Roberts and Roberts (2016), who demonstrated that chimpanzees ( *Pan troglodytes* ) use vocalizations to maintain kin relationships and strengthen group cohesion. Putting together, these results emphasize that vocalization behavior in Tibetan macaques is shaped by both biological and social factors, facilitating group integration.

Our results indicate that ecological factors such as wind speed, temperature, and humidity significantly influenced the vocal activity level of Tibetan macaques. These effects were consistent with patterns reported during the daytime, suggesting that environmental drivers of vocal activity extend into nocturnal periods. While the ACI provides an indirect measure of acoustic activity (Wilson et al. 2015), we acknowledge its limitations, particularly its sensitivity to non‐biological noise such as wind interference. Therefore, interpretations of environmental effects on vocal patterns should be approached with caution.

Furthermore, Tibetan macaque vocalization behavior is closely tied to their social context. Previous studies have documented the vocal repertoire of Tibetan macaques during the daytime and described the acoustic structure and contexts of several call types (Bernstein et al. 2016). Our findings extend them by demonstrating how nocturnal vocalizations are embedded in specific social and ecological contexts. Our study revealed that vocalizations peak during evening hours, when social and aggressive behaviors are frequent, possibly due to competition for sleeping sites and the maintenance of social relationships within the group. This finding aligns with research on other primates, suggesting that vocalizations serve not only to transmit information but also as a crucial tool for maintaining group cohesion and mitigating conflict. For example, studies on chimpanzees have shown that vocalizations often accompany post‐conflict reconciliation behaviors, helping to restore group stability and reduce future conflict likelihood (De Waal and Van Roosmalen 1979; Fuentes et al. 2002). Research on long‐tailed macaques ( *Macaca fascicularis* ) also shows that individuals use vocalizations after aggressive encounters to reconcile and repair social relationships (Cords 1992). Similarly, gibbon vocalizations during chorusing behavior play a dual role in territorial defense and strengthening social bonds, promoting group stability and integration (Geissmann 2002). These findings suggest that even at night, when many primates are less socially active, vocalizations remain a vital tool for reinforcing social bonds and maintaining group integrity. In fact, primates often vocalize during social separation, highlighting the importance of vocalizations in alleviating anxiety and maintaining relationships. For instance, in studies on squirrel monkeys ( *Saimiri sciureus* ), it has been found that vocalizations during periods of separation help monkeys reduce emotional stress and maintain connections with key social partners (Jürgens 1979).

In conclusion, our study underscores the temporal patterns and activity level of nocturnal vocalizations in Tibetan macaques, with two distinct peaks observed at 18:00–19:00 and 22:00–23:00. Both social‐biological and ecological factors contributed to shaping vocal activity patterns. Notably, our findings suggest that even under relatively low temperature and high humidity conditions, Tibetan macaques maintain or even increase vocal activity, potentially reflecting enhanced social cohesion and adaptive strategies during challenging environmental periods. While ACI offers a useful proxy for vocal activity, its limitations require cautious interpretation. Future studies may benefit from detailed analysis of specific vocal types and their associated behavioral contexts to further elucidate the function of nocturnal vocalizations.

## Author Contributions

**Xin Gao:** conceptualization (equal), data curation (equal), formal analysis (equal), investigation (equal), methodology (equal), project administration (equal), resources (equal), software (equal), supervision (equal), validation (equal), visualization (equal), writing – original draft (equal), writing – review and editing (equal). **Tong Zhang:** data curation (equal), formal analysis (equal), investigation (equal), methodology (equal), supervision (equal), visualization (equal), writing – original draft (equal), writing – review and editing (equal). **Jingjing Wang:** data curation (equal), investigation (equal), project administration (equal), supervision (equal). **Dongxin Yang:** formal analysis (equal), software (equal), supervision (equal), visualization (equal). **Xue Chen:** data curation (equal), project administration (equal), software (equal), supervision (equal). **Peipei Yang:** data curation (equal), formal analysis (equal), investigation (equal), methodology (equal). **Wenbo Li:** methodology (equal), project administration (equal), resources (equal), supervision (equal). **Binghua Sun:** formal analysis (supporting), investigation (supporting), methodology (supporting), supervision (supporting). **Dongpo Xia:** investigation (equal), supervision (equal). **Jinhua Li:** conceptualization (supporting), funding acquisition (lead), methodology (supporting), resources (supporting), supervision (supporting). **Xi Wang:** conceptualization (equal), data curation (supporting), formal analysis (equal), funding acquisition (lead), investigation (supporting), methodology (equal), project administration (equal), resources (supporting), supervision (supporting), writing – review and editing (equal).

## Funding

This research was supported by the National Natural Science Foundation of China (Grant numbers: 32370535, 31801983), the Key Project for Natural Science Research in Universities of Anhui Provincial Department of Education of China (Grant number: 2022AH050114), and China Scholarship Council.

## Conflicts of Interest

The authors declare no conflicts of interest.

## Supporting information


**Table S1:** Variance inflation factor (VIF) values for predictor variables in the GLMM analysis.

**Table S2:** Variance inflation factor (VIF) for explanatory variables in the generalized additive model (GAM).

## Data Availability

Derived data supporting the findings of this study have been deposited in the Dryad database and the data can be accessed at https://doi.org/10.6084/m9.figshare.30846914. Additional data related to this study may be requested from the corresponding author upon reasonable request.
